# Pain, Sleep Latency, and Mental and Physical Health in Individuals with Self-Reported Hypermobile Ehlers-Danlos Syndrome

**DOI:** 10.3390/healthcare14111573

**Published:** 2026-06-04

**Authors:** Frank Tudini, Caitlin Crews-Stowe, David Levine

**Affiliations:** 1 Physical Therapy, University of Tennessee at Chattanooga, Chattanooga, TN 37403, USA; david-levine@utc.edu; 2 Health and Human Performance, University of Tennessee at Chattanooga, Chattanooga, TN 37403, USA; caitlin-crews-stowe@utc.edu

**Keywords:** hypermobile Ehlers-Danlos syndrome, chronic pain, pain frequency, sleep disturbance, sleep latency, mental health, physical health, health burden

## Abstract

**Background:** Individuals with hypermobile Ehlers–Danlos syndrome (hEDS) frequently report chronic pain, sleep-related complaints, and reduced mental and physical health. While sleep disturbance has been described in this population, less is known about how pain frequency relates to sleep initiation and broader health burden. This study aimed to examine the associations between pain frequency, sleep latency, and mental and physical health burden in individuals with hEDS. **Methods:** This analysis used cross-sectional survey data collected from adults with self-reported EDS. Pain frequency was treated as an ordinal variable. Sleep disturbance was assessed via self-reported sleep latency, and mental and physical health burden were defined as reporting 14 or more days of poor health in the prior 30 days. Ordinal logistic regression was used to examine associations between pain frequency and sleep latency, adjusting for age group. Binary logistic regression models examined associations between pain frequency and mental and physical health burden. **Results:** A total of 2365 respondents with self-reported hEDS were included in the analysis. Increasing pain frequency was associated with progressively longer sleep latency, demonstrating a clear dose–response relationship (*p* < 0.001). Greater pain frequency was also independently associated with increased odds of reporting frequent poor mental health days (OR = 1.35, 95% CI [1.26, 1.45], *p* < 0.001) and poor physical health days (OR = 1.82, 95% CI [1.66, 1.99], *p* < 0.001). **Conclusions:** Pain frequency was significantly associated with longer sleep latency and overall health burden in individuals with hEDS. These findings suggest that pain and sleep disturbance are interconnected components of a broader symptom burden rather than isolated clinical features. Routine assessment of sleep disturbance in individuals with frequent pain may help identify those at greater risk for broader health burden.

## 1. Introduction

Ehlers–Danlos syndrome (EDS) comprises a heterogeneous group of heritable connective tissue disorders characterized by joint hypermobility, skin hyperextensibility, and tissue fragility [1,2]. Individuals with EDS frequently experience chronic pain, fatigue, and reduced health-related quality of life, with symptoms often emerging early in life and persisting across the lifespan [3,4]. Among the various EDS subtypes, hypermobile EDS (hEDS) is the most commonly reported and is particularly associated with chronic musculoskeletal pain and functional limitations [5].

Sleep disturbance is a common and clinically meaningful concern in adolescents and adults with EDS; however, much of the existing literature has focused on pediatric, adolescent, or mixed-age samples, leaving adult symptom relationships less understood. Prior studies have documented reduced sleep quality, prolonged sleep latency, decreased sleep efficiency, and a high prevalence of sleep disorders—including insomnia, obstructive sleep apnea, and periodic limb movement disorder—particularly in pediatric and young adult populations with hEDS [6,7]. Sleep disruption in EDS has also been associated with poorer health-related quality of life and greater psychiatric comorbidity, underscoring the potential role of sleep as more than a secondary symptom of the condition [8,9].

Pain and sleep disturbance are widely described in the broader literature as having a bidirectional relationship. Chronic pain can disrupt sleep initiation and maintenance, while insufficient or poor-quality sleep has been shown to lower pain thresholds, increase pain sensitivity, and exacerbate symptom perception. Experimental and clinical studies suggest that sleep disruption can impair endogenous pain-modulating systems, alter neuroendocrine function, and contribute to central sensitization [10,11]. These mechanisms are particularly relevant in EDS, where chronic pain, autonomic dysfunction, and neuroinflammatory processes are commonly reported [12,13].

Beyond sleep disturbance, chronic pain has well-established associations with both mental and physical health outcomes. Individuals with persistent pain are more likely to experience anxiety, depression, psychological distress, and reduced physical functioning. Importantly, pain, sleep disturbance, and mental health symptoms often co-occur and appear to exert mutually reinforcing effects, contributing to variability in symptom severity and overall health burden [14,15]. In younger populations, chronic pain has been associated with an increased risk of developing mental health conditions, suggesting the need for early identification and integrated management strategies [15].

Although broader sleep-related complaints have been documented in hEDS, the relationship between pain frequency and sleep initiation and its downstream association with overall health burden remains poorly understood [6,16]. Conceptualizing pain, sleep disturbance, and health outcomes as clinically interrelated components of symptom burden may help explain variability in functional outcomes and identify clinically relevant targets for screening and intervention.

Therefore, the purpose of this exploratory secondary analysis was to examine the relationships between pain frequency, sleep latency, and mental and physical health burden in individuals with hEDS. Specifically, we examined whether greater pain frequency was associated with longer sleep latency and increased odds of reporting frequent poor mental and physical health days. Based on prior literature, we anticipated that pain frequency would demonstrate a dose–response relationship with sleep latency and would be associated with both mental and physical health burden.

## 2. Materials and Methods

### 2.1. Study Design and Participants

This study represents an exploratory secondary analysis of cross-sectional survey data previously collected to examine sleep characteristics in individuals with hEDS. The original study design, survey development, and recruitment procedures have been described in detail elsewhere [6]. Briefly, an anonymous electronic survey was distributed through the Ehlers–Danlos Society website to individuals with a self-reported diagnosis of EDS. The original survey did not include independent medical record verification, specialist confirmation, or additional screening questions regarding the provider who established the hEDS diagnosis; therefore, diagnostic classification relied entirely on participant self-report. For the present analysis, the sample was restricted to respondents who reported a diagnosis of hEDS, which comprised approximately 93% of the original cohort. Because this study was a secondary analysis of an existing survey dataset, no a priori sample size calculation was performed. The final sample reflected all eligible respondents with complete data for the relevant analyses.

Participants were eligible if they were 18 years of age or older and reported a diagnosis of Ehlers–Danlos syndrome. Participation was voluntary, and electronic informed consent was obtained prior to survey initiation. Responses were collected anonymously using QuestionPro (QuestionPro Inc., Seattle, WA, USA). To minimize duplicate responses, digital fingerprinting was used to identify and remove potential duplicate entries prior to analysis. The full survey instrument is provided as Supplementary Material (File S1). Full ordinal regression model parameters are provided in Supplementary Table S1. The study was reviewed by the University of Tennessee at Chattanooga Institutional Review Board and determined to be exempt (IRB #21-133).

### 2.2. Measures

#### 2.2.1. Pain Frequency

Pain frequency was assessed using a self-report item asking respondents how often they experienced pain over the previous seven days. Response options included: never, less than once a week, once or twice a week, three or four times a week, five or six times a week, and every day. Responses were treated as an ordinal variable, with higher values reflecting greater pain frequency. Pain frequency served as the primary independent variable for all analyses in the present study.

#### 2.2.2. Sleep Latency

Sleep latency was assessed using a single self-report survey item asking respondents to estimate the typical time (in minutes) required to fall asleep. This item was selected as part of the original sleep survey because delayed sleep initiation is a clinically relevant and commonly reported sleep complaint in individuals with hEDS and served as the primary sleep-related variable for secondary analysis.

#### 2.2.3. Mental and Physical Health Burden

Mental and physical health burden were assessed using items adapted from the Centers for Disease Control and Prevention Healthy Days Measures, which ask respondents to report the number of days in the past 30 days during which their mental or physical health was “not good.” For analysis, responses were dichotomized at ≥14 days per month, to reflect substantial health burden, consistent with Centers for Disease Control and Prevention Healthy Day Measures [17]. Separate binary outcome variables were created for poor mental health days and poor physical health days.

#### 2.2.4. Covariates

Age was categorized into seven predefined groups (18–24, 25–34, 35–44, 45–54, 55–64, 65–74, and ≥75 years) based on the original survey structure and included as an ordinal covariate in all regression models. Age was included based on prior literature demonstrating age-related differences in pain perception, sleep, and health outcomes in adults with chronic pain and hEDS [18,19,20]. Additional covariates of potential clinical relevance were not consistently available in the original dataset and were therefore not included in the present secondary analysis. Sex and EDS subtype distributions were described descriptively but were not included as covariates due to limited variability (predominantly female sample), and the focus of the present analysis.

### 2.3. Statistical Analysis

All statistical analyses were conducted using IBM SPSS Statistics (Version 29). Due to missing survey responses, the number of participants included varied slightly across models. Descriptive statistics were used to characterize the study sample and pain frequency distribution.

Ordinal logistic regression was used to examine the association between pain frequency and sleep latency, adjusting for age group. The proportional odds assumption was evaluated using the test of parallel lines prior to interpretation of the model. Binary logistic regression models were used to examine the association between pain frequency and (1) mental health burden and (2) physical health burden, each defined as reporting 14 or more days per month of poor health. Pain frequency was modeled as an ordinal predictor in all analyses, with age group included as a covariate.

Model fit was assessed using likelihood ratio chi-square tests and pseudo R^2^ statistics where appropriate. Odds ratios (ORs) with corresponding *p*-values were reported for logistic regression analyses. Statistical significance was set a priori at *p* < 0.05.

## 3. Results

### 3.1. Sample Characteristics

A total of 2365 individuals with self-reported hEDS were included in this analysis. The sample was predominantly female and distributed across adult age groups, with the largest proportion between 25 and 54 years of age. Pain frequency was high across the sample, with over one-third of respondents reporting pain every day. Detailed demographic and clinical characteristics of the study sample are presented in Table 1. A substantial proportion of respondents reported frequent pain, with 36.4% indicating daily pain and the majority reporting pain at least several times per week, supporting pain frequency as a clinically relevant exposure for the present analysis.

### 3.2. Pain and Sleep Latency

An ordinal logistic regression model was used to examine the association between pain frequency and sleep latency, adjusting for age group. The overall model was statistically significant (χ^2^(6) = 244.60, *p* < 0.001). The proportional odds assumption was met (test of parallel lines, *p* = 0.171), supporting the use of the ordinal logistic model. Increasing pain frequency was associated with progressively longer sleep latency. A clear dose–response relationship was observed, with higher pain frequency corresponding to increased odds of longer time to fall asleep (all *p* < 0.001). Age group was also significantly associated with sleep latency, with older age groups demonstrating lower odds of prolonged latency (*p* < 0.001). Full characterization of the analysis can be found in Table 2.

Pain frequency was modeled as an ordinal predictor. Full ordinal regression parameter estimates, including threshold and location parameters, are provided in Supplementary Table S1. Model fit: χ^2^(6) = 244.60, *p* < 0.001; McFadden R^2^ = 0.027.

### 3.3. Pain and Mental Health

A binary logistic regression model was used to examine the association between pain frequency and mental health burden, defined as reporting 14 or more days per month of poor mental health. The overall model was statistically significant (χ^2^(2) = 136.62, *p* < 0.001), explaining approximately 7.7% of the variance in mental health burden (Nagelkerke R^2^ = 0.077). Greater pain frequency was associated with significantly increased odds of reporting frequent poor mental health days (OR = 1.35, *p* < 0.001). This corresponds to a 35% increase in odds of frequent poor mental health days for each incremental increase in pain frequency. Increasing age was associated with decreased odds of reporting poor mental health burden (OR = 0.78, *p* < 0.001). Full characterization of the analysis can be found in Table 3.

### 3.4. Pain and Physical Health

A binary logistic regression model was conducted to examine the association between pain frequency and physical health burden, defined as reporting 14 or more days per month of poor physical health. The overall model was statistically significant (χ^2^(2) = 195.53, *p* < 0.001), explaining approximately 12.6% of the variance in physical health burden (Nagelkerke R^2^ = 0.126). Greater pain frequency was associated with significantly increased odds of reporting frequent poor physical health days (OR = 1.82, *p* < 0.001), representing an 82% increase in odds. Increasing age was associated with slightly decreased odds of reporting poor physical health burden (OR = 0.90, *p* = 0.004). Full characterization of the analysis can be found in Table 4.

These findings indicate that greater pain frequency was associated with longer sleep latency and increased mental and physical health burden in individuals with hEDS.

## 4. Discussion

The purpose of this exploratory analysis was to examine the relationships between pain frequency, sleep latency, and mental and physical health burden in individuals with self-reported hEDS. The findings indicate that higher pain frequency was independently associated with longer sleep latency and poorer mental and physical health. These results support the idea that pain and sleep disturbance in hEDS may represent clinically interconnected symptoms rather than isolated clinical features.

### 4.1. Pain and Sleep Disturbance

A key finding of this study was the strong, dose–response relationship between pain frequency and sleep latency. Individuals reporting more frequent pain were progressively more likely to experience prolonged time to fall asleep, even after adjusting for age. This is consistent with prior literature demonstrating that pain disrupts sleep initiation and continuity across a range of musculoskeletal and chronic pain conditions [21,22]. Nighttime pain has been shown to interfere with sleep quality and latency in populations with rotator cuff pathology and other painful conditions, with higher pain intensity associated with worse sleep disturbance [23,24]. Although the present study did not assess the timing of pain symptoms, the prior literature suggests that pain occurring during evening or nighttime hours may be particularly disruptive to sleep initiation. Our findings support a broader association between pain frequency and delayed sleep initiation in individuals with hEDS. From a clinical perspective, sleep latency exceeding approximately 30 min is commonly considered indicative of difficulty initiating sleep in adult sleep literature. Although the present study used broader categorical ranges based on the original survey design rather than diagnostic thresholds, a substantial proportion of respondents reported sleep latency beyond this clinically meaningful range. This interpretation should be considered within the context of the diagnostic complexity of hEDS, where overlap with hypermobility spectrum disorders, dysautonomia, fatigue syndromes, and other chronic pain presentations are common.

Prior experimental and clinical studies suggest that disrupted sleep may be associated with heightened pain sensitivity, altered nociceptive processing, and reduced efficiency of endogenous pain modulation. These mechanisms may be relevant to individuals with hEDS, although the present cross-sectional design does not allow direct mechanistic or causal inference [11,25,26,27]. The present results suggest that difficulty initiating sleep may help clinicians identify individuals reporting broader symptom burden in this population.

From an hEDS-specific perspective, these findings may reflect the broader multisystem nature of the condition. Individuals with hEDS commonly report autonomic dysfunction, fatigue, connective tissue instability, gastrointestinal complaints, and heightened sensory sensitivity, all of which may contribute to difficulty initiating sleep and maintaining symptom control across the lifespan. Dysautonomia and overlapping chronic pain presentations may further amplify symptom burden in this population. Emerging literature has also proposed neuroinflammatory and central sensitization mechanisms as potential contributors to persistent symptoms, although these mechanisms were not directly examined in the present study.

### 4.2. Pain, Mental Health, and Physical Health Burden

Pain frequency was also strongly associated with both mental and physical health burden, defined as frequent poor health days over the prior month. Each increase in pain frequency was associated with substantially higher odds of reporting frequent poor physical health days, with a more modest but still significant association observed for mental health burden. These findings align with prior work demonstrating that chronic pain is a major contributor to functional limitation, reduced quality of life, and psychological distress [28,29].

The stronger association observed between pain frequency and physical health burden may reflect the direct impact of persistent pain on mobility, fatigue, and daily functioning in individuals with hEDS. At the same time, the association between pain frequency and mental health burden supports prior evidence that chronic pain and mental health conditions, including anxiety and depression, frequently co-occur and may exert mutually reinforcing effects [30]. Neuroinflammatory processes, maladaptive neuroplasticity, and chronic stress responses have been proposed as shared mechanisms underlying pain persistence and psychological distress, and these mechanisms may be particularly relevant in hEDS [31,32].

Age was inversely associated with both mental and physical health burden in the present analyses, consistent with findings from the primary study and prior literature [6,33,34]. This pattern may reflect adaptive coping strategies, altered symptom appraisal, survivorship effects, or age-related changes in symptom presentation over time. However, longitudinal research is needed to better understand these relationships in individuals with hEDS.

Although the regression models were statistically significant, the pseudo-R^2^ values suggest that pain frequency and age explained only a modest proportion of the variance in sleep latency and perceived health burden. This is not unexpected in individuals with hEDS, where symptom presentation is often influenced by multiple interacting biological, psychological, and behavioral factors. These findings suggest that pain frequency may represent one clinically relevant contributor, but not the sole driver, of sleep-related and health-related outcomes in this population.

### 4.3. Clinical Implications

These findings support contemporary multidimensional models of chronic illness, in which symptoms such as pain, sleep disturbance, and psychological distress are closely interconnected rather than hierarchically organized. From a clinical perspective, these findings support routine assessment of both pain frequency and sleep initiation in individuals with hEDS, particularly in those presenting with persistent fatigue, widespread pain, reduced physical activity tolerance, or declining mental health. Although sleep latency alone does not capture all dimensions of sleep health, difficulty initiating sleep may represent a clinically relevant feature associated with broader symptom burden in this population. Clinicians—including physical therapists, physicians, psychologists, and sleep specialists—may benefit from incorporating brief sleep-related screening questions alongside pain and functional assessment, particularly when recovery is slower than expected or symptom irritability remains high.

Importantly, sleep should not be viewed solely as a secondary consequence of pain in individuals with hEDS. Emerging evidence suggests that disrupted sleep may be associated with heightened pain sensitivity and psychological distress, reinforcing the need for integrated, multidisciplinary approaches to care. Behavioral sleep interventions, pain management strategies, autonomic regulation, graded physical conditioning, and psychosocial support may all play complementary roles in reducing overall symptom burden in this population [35,36].

### 4.4. Limitations

Several limitations should be considered when interpreting the findings of this study. First, the data were obtained through a self-reported, cross-sectional survey, which may be subject to recall bias and misclassification despite restricting the sample to individuals with hEDS. Pain frequency, sleep latency, and mental and physical health burden were not confirmed through objective clinical assessment or validated diagnostic interviews, and respondents may have over- or underestimated symptom severity. In addition, sleep was operationalized using self-reported sleep latency alone. Although sleep latency represents a clinically relevant aspect of sleep health, it does not capture other important dimensions such as sleep duration, sleep efficiency, nighttime awakenings, daytime dysfunction, or overall insomnia severity. As a result, the present findings should be interpreted specifically in relation to sleep initiation rather than overall sleep quality. In addition, the survey assessed pain frequency but did not capture the timing of pain symptoms (e.g., daytime versus evening or nighttime pain). Because pain occurring later in the day may have a more direct influence on sleep initiation, the absence of temporal pain data may have reduced precision in interpreting the relationship between pain frequency and sleep latency.

Second, the cross-sectional design precludes causal inference regarding the directionality of relationships among pain, sleep disturbance, and health outcomes. Although the findings are consistent with a bidirectional pain–sleep relationship described in prior literature [37], longitudinal studies are needed to clarify temporal pathways and determine whether sleep disturbance mediates or amplifies pain-related health burden in individuals with Ehlers–Danlos syndrome.

Third, Ehlers–Danlos syndrome diagnoses were self-reported and were not independently confirmed using the 2017 international diagnostic criteria for hEDS. As a result, some participants may have met criteria for related conditions such as hypermobility spectrum disorders or other overlapping chronic pain or fatigue presentations rather than hEDS alone. Given the known clinical overlap among hEDS, chronic pain syndromes, autonomic dysfunction, and fatigue-related conditions, some degree of diagnostic misclassification is possible and may have influenced internal validity. In addition, our study sample was predominantly female and largely composed of individuals reporting hEDS, which may limit generalizability to other EDS subtypes and more diverse populations.

Fourth, although age was included as a covariate based on prior literature, the present analysis did not account for several other clinically relevant factors that may influence pain, sleep latency, and perceived health burden in individuals with hEDS. These may include medication use (e.g., analgesics or sleep medications), psychiatric comorbidity, autonomic dysfunction, fatigue severity, physical activity levels, pain intensity, pain duration, and other medical comorbidities. As a result, residual confounding is likely, and the observed associations should be interpreted as exploratory rather than fully adjusted estimates of independent relationships.

Finally, selection bias is possible, as participants were recruited through the Ehlers-Danlos Society website and may represent individuals with greater symptom burden, higher health engagement, or increased interest in sleep- and pain-related concerns. This recruitment strategy may have contributed to higher reported symptom prevalence and may have strengthened observed associations compared with a more broadly sampled hEDS population. As with many survey-based studies, the findings may therefore not fully represent the broader population of individuals with Ehlers–Danlos syndrome.

### 4.5. Conclusions

This analysis demonstrates that pain frequency is significantly associated with longer sleep latency and both mental and physical health burden in individuals with hEDS. Increasing pain frequency was associated with longer sleep latency and substantially higher odds of reporting frequent poor physical health and mental health days. These findings suggest that pain and sleep disturbance represent interconnected components of a broader symptom burden rather than isolated clinical features in hEDS. From a clinical perspective, pain frequency—not just intensity—may help clinicians identify individuals with hEDS who are more likely to report delayed sleep initiation and broader health burden. Future longitudinal studies are needed to clarify causal pathways and to determine whether interventions targeting sleep disturbance can mitigate pain-related health outcomes in this population.

## Figures and Tables

**Table 1:** Sample characteristics of participants with self-reported hEDS (N = 2365).

Characteristic	n (%)
Gender	
Male	80 (3.4)
Female	2216 (93.7)
Prefer not to answer	60 (2.5)
Age Group	
18–24	328 (13.9)
25–34	551 (23.3)
35–44	619 (26.2)
45–54	426 (18.0)
55–64	313 (13.2)
65–74	109 (4.6)
≥75	13 (0.5)
Pain Frequency	
Never	49 (2.1)
Less than once a week	163 (6.9)
Once or twice a week	392 (16.6)
Three or four times a week	486 (20.5)
Five or six times a week	408 (17.3)
Every day	857 (36.2)
Sleep Latency	
0–15 min	340 (14.4)
16–30 min	429 (18.1)
31–45 min	318 (13.4)
46–60 min	390 (16.5)
61–90 min	379 (16.0)
91–120 min	254 (10.7)
>120 min	242 (10.2)

Note: Percentages are based on the total study sample. Category totals vary slightly due to missing survey responses.

**Table 2:** Association between pain frequency and sleep latency in individuals with hypermobile Ehlers–Danlos Syndrome.

Predictor	Estimate (B)	SE	Wald χ^2^	*p*-Value	95% CI
Pain frequency (overall ordinal effect)	—	—	—	<0.001	—
Age group	−0.232	0.026	78.38	<0.001	−0.283, −0.180

**Table 3:** Logistic regression predicting poor mental health burden from pain frequency.

Predictor	B	SE	Wald χ^2^	OR (Exp(B))	*p*-Value
Pain frequency	0.301	0.036	70.51	1.35	<0.001
Age group	−0.254	0.031	66.11	0.78	<0.001

OR = odds ratio. Model fit: χ^2^(2) = 136.62, *p* < 0.001; Nagelkerke R^2^ = 0.077.

**Table 4:** Logistic regression predicting poor physical health burden from pain frequency.

Predictor	B	SE	Wald χ^2^	OR (Exp(B))	*p*-Value
Pain frequency	0.596	0.046	170.36	1.82	<0.001
Age group	−0.104	0.036	8.11	0.90	0.004

OR = odds ratio. Model fit: χ^2^(2) = 195.53, *p* < 0.001; Nagelkerke R^2^ = 0.126.

## Data Availability

The raw data supporting the conclusions of this article will be made available by the authors on request.

## Supplementary Materials

The following supporting information can be downloaded at: https://www.mdpi.com/article/10.3390/healthcare14111573/s1, File S1. Survey. Table S1. Full Ordinal Logistic Regression Model Parameters for the Association Between Pain Frequency and Sleep Latency.
