# Supplementary material for: Pain, Sleep Latency, and Mental and Physical Health in Individuals with Self-Reported Hypermobile Ehlers-Danlos Syndrome
Source: Healthcare (Basel). 2026 Jun 4;14(11):1573. doi: 10.3390/healthcare14111573 (PMC13257359; doi:10.3390/healthcare14111573)
Supplement: Supplementary file 1 [file healthcare-14-01573-s001.zip › Supplementary Table S1.pdf]

# Supplementary Table S1. Full Ordinal Logistic Regression Model Parameters for the Association Between Pain Frequency and Sleep Latency

| Parameter | Estimate (B) | SE | Wald $\chi^2$ | df | p-value | 95% CI |
|---|---|---|---|---|---|---|
| **Threshold Parameters** | | | | | | |
| 0–15 minutes | -2.362 | 0.498 | 22.449 | 1 | <0.001 | -3.339 to -1.385 |
| 16-30 minutes | -1.236 | 0.497 | 6.195 | 1 | 0.013 | -2.209 to -0.263 |
| 31-45 minutes | -0.617 | 0.496 | 1.547 | 1 | 0.214 | -1.589 to 0.355 |
| 46-60 minutes | 0.115 | 0.496 | 0.053 | 1 | 0.817 | -0.857 to 1.086 |
| 61-90 minutes | 0.964 | 0.496 | 3.774 | 1 | 0.052 | -0.009 to 1.937 |
| 91-120 minutes | 1.846 | 0.498 | 13.722 | 1 | <0.001 | 0.869 to 2.823 |
| **Pain Frequency Parameters** *(Reference = Pain every day)* | | | | | | |
| Never | -1.814 | 0.267 | 46.219 | 1 | <0.001 | -2.337 to -1.291 |
| Less than once a week | -1.496 | 0.155 | 93.117 | 1 | <0.001 | -1.800 to -1.192 |
| Once or twice a week | -1.039 | 0.109 | 90.068 | 1 | <0.001 | -1.253 to -0.824 |

| Parameter | Estimate (B) | SE | Wald $\chi^2$ | df | p-value | 95% CI |
|---|---|---|---|---|---|---|
| Three or four times a week | -0.690 | 0.101 | 46.579 | 1 | <0.001 | -0.888 to -0.492 |
| Five or six times a week | -0.507 | 0.106 | 22.707 | 1 | <0.001 | -0.715 to -0.298 |
| **Age Group Parameters** *(Reference = Age ≥75 years)* | | | | | | |
| 18–24 years | 0.674 | 0.500 | 1.814 | 1 | 0.178 | -0.307 to 1.654 |
| 25–34 years | 0.309 | 0.497 | 0.388 | 1 | 0.534 | -0.664 to 1.283 |
| 35–44 years | 0.132 | 0.496 | 0.070 | 1 | 0.791 | -0.841 to 1.104 |
| 45–54 years | -0.301 | 0.499 | 0.364 | 1 | 0.546 | -1.278 to 0.676 |
| 55–64 years | -0.186 | 0.501 | 0.138 | 1 | 0.710 | -1.169 to 0.796 |
| 65–74 years | -0.659 | 0.520 | 1.603 | 1 | 0.205 | -1.678 to 0.361 |

**Abbreviations:** B = regression coefficient; SE = standard error; CI = confidence interval.

**Note:** Threshold parameters represent cumulative cut-points across ordered sleep latency categories. Pain frequency and age group were entered into the model as categorical predictors using indicator coding. Reference categories were *pain every day* for pain frequency and *≥75 years* for age group. The proportional odds assumption was met (test of parallel lines, $p = 0.171$).
